# The metabolic footprint of *Clostridia* and *Erysipelotrichia* reveals their role in depleting sugar alcohols in the cecum

**DOI:** 10.1186/s40168-021-01123-9

**Published:** 2021-08-19

**Authors:** Connor R. Tiffany, Jee-Yon Lee, Andrew W. L. Rogers, Erin E. Olsan, Pavel Morales, Franziska Faber, Andreas J. Bäumler

**Affiliations:** 1 Department of Medical Microbiology and Immunology, School of Medicine, University of California, Davis, One Shields Avenue, Davis, CA 95616 USA; 2 Present Address: Department of Biological Sciences, California State University Sacramento, 6000 J Street, Sacramento, CA 95819 USA; 3 Department of Pathology, Microbiology and Immunology, School of Veterinary Medicine, University of California, Davis, One Shields Avenue, Davis, CA 95616 USA; 4 Present Address: Institute for Molecular Infection Biology (IMIB), Faculty of Medicine, University of Würzburg, Josef-Schneider-Street 2/D15, 97080 Würzburg, Germany

**Keywords:** Microbiota, Alcoholic sugars, Polyols, FODMAPs, *Clostridia*, *Erysipelotrichia*

## Abstract

**Background:**

The catabolic activity of the microbiota contributes to health by aiding in nutrition, immune education, and niche protection against pathogens. However, the nutrients consumed by common taxa within the gut microbiota remain incompletely understood.

**Methods:**

Here we combined microbiota profiling with an un-targeted metabolomics approach to determine whether depletion of small metabolites in the cecum of mice correlated with the presence of specific bacterial taxa. Causality was investigated by engrafting germ-free or antibiotic-treated mice with complex or defined microbial communities.

**Results:**

We noted that a depletion of *Clostridia* and *Erysipelotrichia* from the gut microbiota triggered by antibiotic treatment was associated with an increase in the cecal concentration of sugar acids and sugar alcohols (polyols). Notably, when we inoculated germ-free mice with a defined microbial community of 14 *Clostridia* and 3 *Erysipelotrichia* isolates, we observed the inverse, with a marked decrease in the concentrations of sugar acids and polyols in cecal contents. The carbohydrate footprint produced by the defined microbial community was similar to that observed in gnotobiotic mice receiving a cecal microbiota transplant from conventional mice. Supplementation with sorbitol, a polyol used as artificial sweetener, increased cecal sorbitol concentrations in antibiotic-treated mice, which was abrogated after inoculation with a *Clostridia* isolate able to grow on sorbitol in vitro*.*

**Conclusions:**

We conclude that consumption of sugar alcohols by *Clostridia* and *Erysipelotrichia* species depletes these metabolites from the intestinal lumen during homeostasis.

Video abstract

**Supplementary Information:**

The online version contains supplementary material available at 10.1186/s40168-021-01123-9.

## Introduction

The gut microbiota plays an important role in the homeostasis and health of its host organism. A large amount of research has focused on how specific constituents of the microbiota interact with the host to influence immune regulation and metabolism [1], but the metabolic capabilities of the gut microbiota remain incompletely understood. Previous research suggests that while phylogeny can be a good predictor of complex metabolic traits requiring large amounts of genes, such as photosynthesis, it is not necessarily a good predictor of simple metabolic traits like carbohydrate utilization [2]. The relationship between phylogeny and metabolic traits in bacteria is complicated further by the widespread occurrence of lateral gene transfer [3], gene loss, and convergent evolution [4]. Nonetheless, some metabolic pathways are conserved among phylogenetic groupings within the gut microbiome [5].

For example, members of the *Enterobacteriaceae*, a family within the phylum *Proteobacteria*, lack the capabilities to break down complex carbohydrates and instead rely on respiratory catabolism of fermentation products using terminal electron acceptors such as nitrate and oxygen [6, 7]. Many *Bacteroides* spp., obligate anaerobes within the phylum *Bacteroidetes*, are primary fermenters of fiber and mucin-derived polysaccharides [8], encoding a large repertoire of genes associated with glycolytic gene clusters [9], while producing much of the short-chain fatty acid propionate found in the murine gut [10]. *Prevotella* spp. within the phylum *Bacteroidetes* can also ferment many plant polysaccharides [11] and the abundance of this genus positively correlates with increased fiber intake in humans [12], but unlike *Bacteroides*, *Prevotella* spp. do not produce propionate [10]. Bacteria within *Clostridia*, a class of spore-forming, obligate anaerobes in the phylum *Firmicutes*, have been linked to the production of the short-chain fatty acid butyrate [13, 14]. This metabolite fulfills an important role in the gut, acting as a signaling molecule and carbon source for host colonic epithelial cells (colonocytes) [15, 16]. By driving colonocyte metabolism towards high oxygen consumption through mitochondrial beta oxidation, butyrate helps maintain the epithelium in a state of physiologic hypoxia [17], thereby preserving anaerobiosis in the lumen of the large bowel [18]. *Clostridia* have also been shown to comprise the bulk of diversity within the human gut microbiome [19], further highlighting the importance of this taxon within the ecosystem of the large intestine. While there is increasing information on the metabolic outputs of *Clostridia* and their function in mediating gut homeostasis, less is known about what nutrients they consume in vivo.

*Clostridia* were recently divided into two classes, *Clostridia* and *Erysipelotrichia*, which form two sister groups within the phylum *Firmicutes* [20]. In light of this change in nomenclature, previous work on butyrate production by *Clostridia* also included *Erysipelotrichia* isolates [21]. Here we combined germ-free mice engrafted with a defined consortium of *Clostridia* and *Erysipelotrichia* isolates with an un-targeted metabolomics approach to determine how these taxa alter the metabolite landscape in the cecum.

## Results

### Streptomycin treatment shifts the cecal metabolome by increasing sugar alcohols and sugar acids

**Fig. 1:**
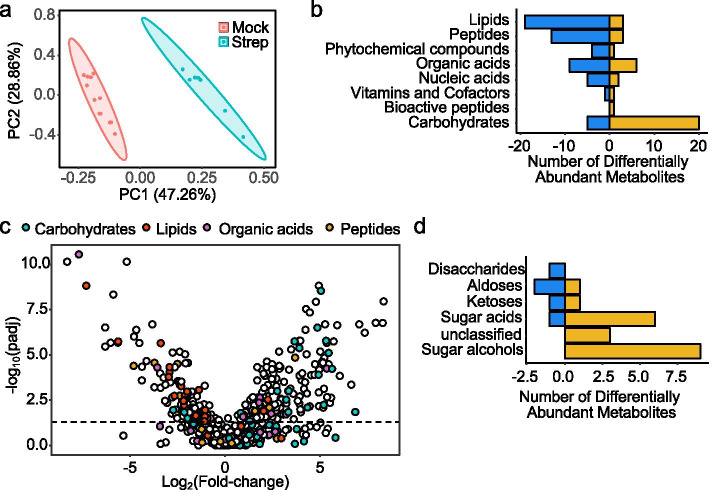
Streptomycin treatment alters the mouse cecal metabolome, characterized by an increase in carbohydrates. Mice were mock-treated (*n* = 12) or received a single dose of streptomycin (*n* = 8) and the cecal metabolome was analyzed 3 days later (mice of each group were housed in three different cages each). **a** PCA plot showing variation of the mouse cecal metabolome by treatment group. **b** Bar plot displaying the number of significantly increased and decreased metabolites by metabolite class in streptomycin-treated mice relative to mock-treated mice (FDR corrected *P* value =  < 0.05). **c** Volcano plot of metabolites colored by class. Metabolites with a positive fold-change value increased with streptomycin treatment while metabolites with a negative-fold change value decreased with streptomycin treatment. The dashed line is set at an FDR corrected *P* value of 0.05. **d** Bar plot displaying the number of differentially significantly increased and decreased carbohydrates by metabolite subclass in streptomycin-treated mice relative to mock-treated mice (FDR corrected *P* value ≤ 0.05)

To investigate how antibiotic-treatment changes the murine cecal metabolome, mice (C57BL/6 J) were mock-treated or received a single dose of streptomycin intragastrically and cecal contents were collected 3 days later. The soluble fraction of the cecal contents was then analyzed by un-targeted gas chromatography time-of-flight mass spectrometry (metabolite profiling). Principle component analysis of the samples showed a distinct clustering of mice by treatment group, indicating that streptomycin treatment changes the composition of the murine cecal metabolome (Fig. 1a and Supplementary Table 1). Consistent with a previous report [22], differential abundance of metabolites between streptomycin-treated mice and mock-treated mice revealed that the concentration of many compounds annotated as carbohydrates significantly increased with streptomycin treatment, while the concentration of many compounds annotated as lipids, peptides, or organic acids decreased (Fig. 1b, c). Interestingly, most of the carbohydrates that increased with streptomycin treatment were identified as sugar acids (galactonic acid, glyceric acid, lactobionic acid, ribonic acid, saccharic acid, threonic acid, and xylonic acid) or sugar alcohols (1,5-anhydroglucitol, erythritol, galacitonol, lyxitol, myo-inositol, pentitol, sorbitol, threitol, and xylitol) (Fig. 1d and Supplementary Table 2). This increase in the amount of sugar alcohols and sugar acids raised the possibility that streptomycin treatment might deplete microbes that normally consume these nutrients in the cecum.

### Streptomycin treatment depletes *Clostridia* and *Erysipelotrichia* from the gut microbiota

**Fig. 2:**
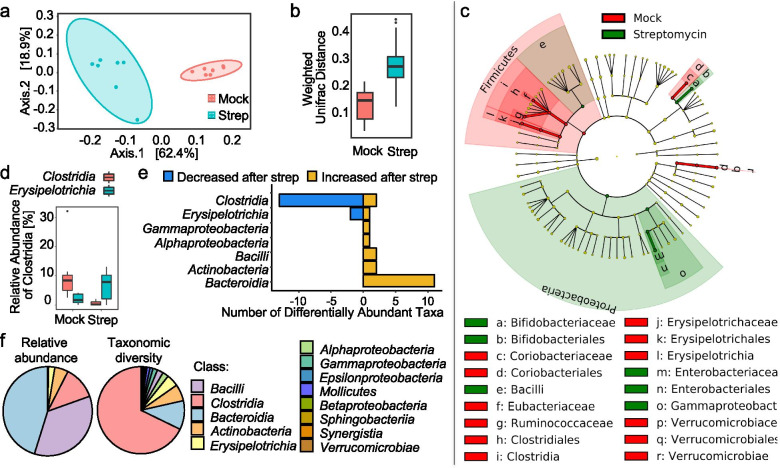
Streptomycin treatment of mice induces a shift in community structure of the large bowel microbiome. Mice were mock-treated (*n* = 8) or received a single dose of streptomycin (*n* = 8) and bacterial communities in the colon contents were analyzed 3 days later. **a** Principal coordinate analysis of 16S rRNA gene amplicon sequencing of colon contents from mice. **b** Box plot of weighted unifrac distance between mock and streptomycin treated mice (*P* value = 0.002). **c** LEFSE cladogram displaying taxa enriched in mock-treated mice (red) or in streptomycin-treated mice (green). Taxa enriched within higher level taxa are indicated by a darker shading. **d** Box plots showing the relative abundance of *Clostridia* and *Erysipelotrichia* in mock-treated and streptomycin-treated mice. **e** Significantly increased and decreased amplicon sequence variants by class in streptomycin-treated mice relative to mock-treated mice (FDR corrected *P* value ≤ 0.05). **f** Relative abundance (left pie chart) and taxonomic diversity of ASVs (right pie chart) in mock-treated mice at the class level

To investigate whether changes in the cecal metabolite profile were linked to specific changes in the community composition, we extracted total genomic DNA from contents of the proximal colon, assembled libraries, and then performed 16S rRNA gene amplicon sequencing (microbiota profiling). Weighted unifrac analysis of the samples showed distinct clustering by treatment group (Fig. 2a), and a significant difference in weighted unifrac distance was observed between streptomycin-treated and mock-treated mice (Fig. 2b), indicating that the community structure was different between groups. *Clostridia* and *Erysipelotrichia* were the only taxonomic groups to both contain significantly decreased amplicon sequence variants (ASVs) in streptomycin-treated mice relative to mock-treated mice according to negative binomial regression analysis, explaining the difference between the gut microbiota of streptomycin-treated mice and mock-treated mice according to linear discriminant analysis (Fig. 2c). Conversely, *Gammaproteobacteria* and *Bacilli* were significantly enriched in streptomycin-treated mice. Consistent with previous reports [18, 23, 24], we observed a decrease in the relative abundance of *Clostridia* in streptomycin-treated mice (Fig. 2d). Within the *Erysipelotrichia*, three taxa dropped below the limit of detection in the streptomycin-treated group, whereas the most abundant taxon significantly increased (Fig. 2e and Fig. S1), resulting in an elevated overall relative abundance of *Erysipelotrichia* in streptomycin-treated mice (Fig. 2d). Differential abundance analysis revealed that the class *Clostridia* had the most significantly different amplicon sequence variants (ASVs), with most of these decreasing after streptomycin treatment (Fig. 2e). Whereas *Clostridia* were not the most abundant taxon, this class comprised approximately two thirds of the ASV diversity within the microbiota of mock-treated mice (Fig. 2f). Thus, streptomycin treatment altered the community structure by depleting *Clostridia* and *Erysipelotrichia* (Fig. 2c–e), with the former being the taxonomically most diverse class within the gut microbiota (Fig. 2f).

### *Clostridia* and *Erysipelotrichia* deplete sugar alcohols and sugar acids

**Fig. 3:**
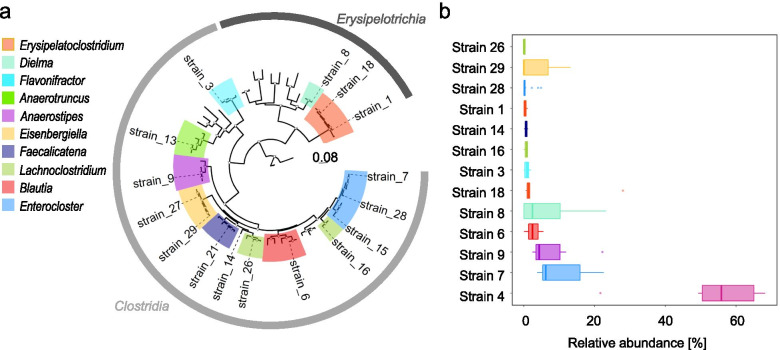
Engraftment of germ-free mice with a defined community of human *Clostridia* and *Erysipelotrichia* isolates. **a** Placement of the members of a defined microbial community (strains 1, 3, 6, 7, 8, 9, 13, 14, 15, 16, 18, 21, 26, 27, 28, and 29) on a maximum likelihood phylogeny built from the alignments of 11 total single copy 30S and 50S ribosomal proteins, which were concatenated together for a total alignment length of 1822 amino acids. The tree encompasses 66 taxa, three of which being *Alicyclobacillus* species belonging to the class *Bacilli* that were included as an outgroup. Strain 4 from the defined microbial community was excluded from analysis because NCBI flagged its deposited genomic sequence as chimeric, containing sequences from two strains belonging to the same species, *Hungatella hathewayi*. Sequences that did not belong to strains within the defined microbial community were obtained from [26]. 150 non-parametric bootstraps were performed to determine confidence of branch placements (white triangle at a node indicate above 70% confidence). **b** Germ-free mice were engrafted with a community of 14 *Clostridia* and 3 *Erysipelotrichia* isolates and microbiota profiling performed on colon contents obtained five days later

**Fig. 4:**
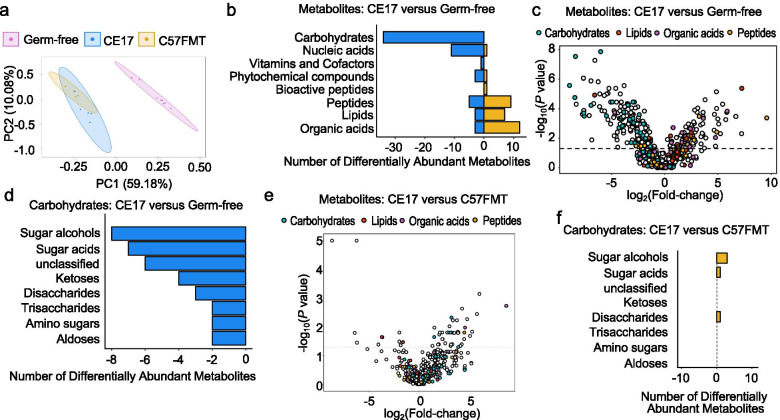
Colonization of germ-free mice with Clostridia induces a shift in the cecal metabolome. Comparative analysis of the cecal metabolome of germ-free mice (*n* = 6), gnotobiotic mice associated with a defined microbial community containing14 human *Clostridia* and 3 human *Erysipelotrichia* isolates (CE17) (*n* = 6), and gnotobiotic mice receiving a cecal microbiota transplant from conventional C57BL/6 J mice (C57FMT) (*n* = 4). **a** Principle component analysis of the cecal metabolome in the indicated groups of mice. **b** Bar plot of significantly increased and decreased metabolites by metabolite class in gnotobiotic mice associated with a defined microbial community (CE17) relative to germ-free mice (FDR corrected *P* value ≤ 0.05). **c** Volcano plot of metabolites colored by class. Metabolites with a positive fold-change value increased in mice engrafted with a defined microbial community compared to germ-free mice, while metabolites with a negative fold-change value decreased. The dashed line is set at an FDR corrected P value of 0.05.** d** Bar plot of significantly increased and decreased carbohydrates by metabolite subclass in gnotobiotic mice associated with a defined microbial community (CE17) relative to germ-free mice (FDR corrected *P* value ≤ 0.05). **e** Volcano plot of metabolites colored by class. Metabolites with a positive fold-change value increased in mice engrafted with a defined microbial community compared to gnotobiotic mice receiving a cecal microbiota transplant from conventional C57BL/6 J mice (C57FMT), while metabolites with a negative fold-change value decreased. **f** Bar plot of significantly increased and decreased metabolites by metabolite class in gnotobiotic mice associated with a defined microbial community (CE17) relative to gnotobiotic mice receiving a cecal microbiota transplant from conventional C57BL/6 J mice (C57FMT) (FDR corrected *P* value ≤ 0.05)

Having established a correlation between *Clostridia* and *Erysipelotrichia* depletion and an increase in sugar alcohols and sugar acids, we next wanted to determine whether *Clostridia* and *Erysipelotrichia* caused this shift in the cecal metabolome in the absence of potential confounding factors, such antibiotic treatment. To that end, we inoculated germ-free Swiss Webster mice with a consortium originally described as 17 commensal *Clostridia* isolated from a healthy volunteer [21]. According to current nomenclature, this consortium consists of fourteen *Clostridia* isolates and three *Erysipelotrichia* isolates [25] (Fig. 3a and Supplementary Table 3). Five days after colonization, samples were collected and analyzed using microbiota and metabolite profiling. Microbiota profiling revealed that ten *Clostridia* isolates and three *Erysipelotrichia* isolates engrafted at levels detectable by 16S ribosomal RNA gene amplicon sequencing (Fig. 3b). Principle component analysis showed a distinct clustering pattern between mock-treated germ-free mice and those given the consortium of commensal *Clostridia* and *Erysipelotrichia* isolates (Fig. 4a). Remarkably, association of germ-free mice with the commensal consortium (Fig. 4b, c and Supplementary Table 4) produced an equivalent but inverse phenotype to that observed after depleting *Clostridia* and *Erysipelotrichia* by treatment with streptomycin (Fig. 1b, c Supplementary Table 1). All significantly different metabolites identified as carbohydrates decreased when germ-free mice were associated with a consortium of commensal *Clostridia* and *Erysipelotrichia* isolates (Fig. 4b, c and Supplementary Table 4), which was accompanied by a concomitant increase in a plurality of significantly different lipids, peptides, and organic acids. Within carbohydrates, sugar alcohols (erythritol, galactinol, lyxitol, myo-inositol, pentitol, sorbitol, ribitol, and xylitol) and sugar acids (digalacturonic acid, galactonic acid, galacturonic acid, glyceric acid, threonic acid, and xylonic acid) contained the largest number of significantly decreased compounds (Fig. 4d and Supplementary Table 5).

Comparing metabolite profiling data from streptomycin-treated mice with those from gnotobiotic mice engrafted with commensal *Clostridia* and *Erysipelotrichia* isolates is complicated by the fact that conventional and gnotobiotic mice differed in their genotype (C57BL/6 J versus Swiss Webster) and the composition of their chow. To compare metabolite profiles in the absence of these confounding variables, germ-free Swiss Webster mice received a cecal microbiota transplant from conventionally housed C57BL/6 J mice. Five days after engraftment, cecal samples were collected and analyzed using metabolite profiling. Principle component analysis revealed clustering of samples from mice receiving a cecal microbiota transplant from conventional (C57BL/6) mice with those from mice engrafted with the consortium of commensal *Clostridia* and *Erysipelotrichia* isolates (Fig. 4a). Notably, only four carbohydrates (including the alcoholic sugars hexitol, pentitol, and threitol) were depleted in germ-free mice receiving a cecal microbiota transplant from conventional (C57BL/6) mice compared to germ-free mice engrafted consortium of commensal *Clostridia* and *Erysipelotrichia* isolates (Fig. 4e, f and Supplementary Table 4). These data suggested that the carbohydrate footprint of a defined microbial community of human *Clostridia* and *Erysipelotrichia* isolates was similar to that produced by a complex mouse microbiota.

### Polyol utilization by *Clostridia *and *Erysipelotrichia* species in vitro and in vivo

**Fig. 5:**
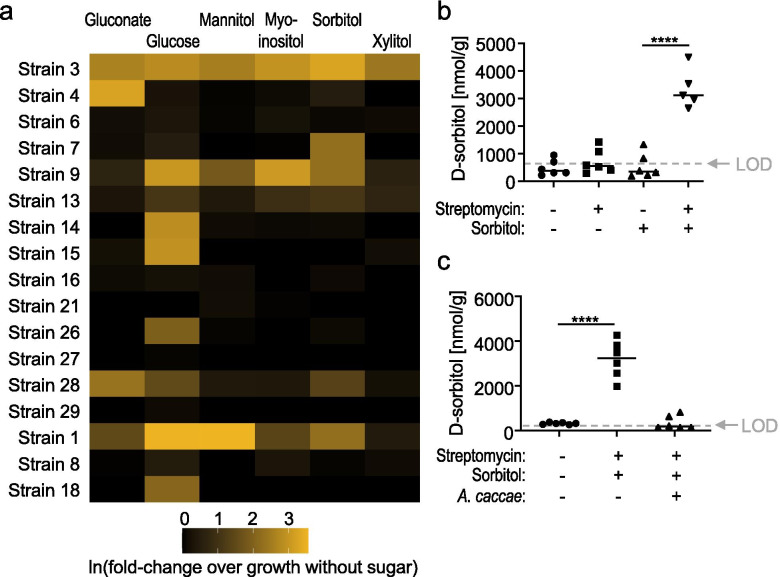
In vivo depletion of sorbitol by sorbitol eaters. **a** Heat map showing growth of individual *Clostridia* and *Erysipelotrichia* isolates on no carbon defined media (NCDM) supplemented with the indicated sugars. **b**, **c** Conventional C57BL/6 J mice were mock treated or treated with a single dose of streptomycin and received drinking water supplemented with or without 5% sorbitol. **b** The concentration of sorbitol in ceca contents was measured by ELISA 3 days after streptomycin treatment. **c** One day after streptomycin treatment, mice were mock inoculated or inoculated with *A. caccae* (designated “strain 9” in panel a)*.* The concentration of sorbitol in cecal contents was measured by a colorimetric assay 3 days after streptomycin treatment. LOD, limit of detection; ****, *P* < 0.0001

This result demonstrated that a consortium of commensal *Clostridia* and *Erysipelotrichia* isolates depletes sugar alcohols and sugar acids in the cecum, but did not provide direct evidence that these bacteria could utilize these carbon sources for growth. Polyols, such as sorbitol, mannitol, or xylitol, are of particular interest, because they are used as artificial sweeteners in a broad range of processed food products. Thus, we performed an in vitro growth assay using no carbon defined media (NCDM) supplemented with D-gluconic, glucose, or one of the following sugar alcohols: xylitol, D-sorbitol, D-mannitol, myo-inositol, and meso-erythritol. Consumption of various combinations of sugar alcohols was observed for six *Clostridia* and one *Erysipelotrichia* isolate, with all seven isolates being able to grow on sorbitol as a carbon source (Fig. 5a).

Sorbitol is commonly used as a supplement to sweeten food. We thus wanted to determine whether consumption of polyols by *Clostridia* could be observed in streptomycin-treated mice receiving dietary sorbitol supplementation. To this end, mice were maintained on a defined diet containing low concentrations of FODMAPs (Teklad Custom Diet TD.110675, Envigo). Mice were then mock-treated or treated with streptomycin and received 5% sorbitol in their drinking water. Absolute sorbitol concentrations determined by a colorimetric assay were close to the limit of detection in cecal contents of mock-treated mice. Sorbitol supplementation did not increase the concentration of sorbitol in cecal contents of mock-treated mice, suggesting that an intact microbiota can deplete this dietary supplement (Fig. 5b). In contrast, disruption of the microbiota by streptomycin treatment resulted in a marked increase in cecal sorbitol levels in mice receiving sorbitol supplementation. To investigate whether consumption of sorbitol by microbes was responsible for depleting this dietary supplement, the experiment was repeated with streptomycin-treated mice that were mock-inoculated or engrafted with *Anaerostipes caccae* (strain 9 from the defined microbial community, originally classified as *Clostridium indolis*) (Fig. 3a and Supplementary Table 3), which was able to grow in vitro using sorbitol as a carbon source (Fig. 5a). Notably, engraftment of streptomycin-treated mice with *A. caccae* resulted in a marked depletion of sorbitol (Fig. 5c), thus further supporting the idea that consumption of polyols by *Clostridia* results in a depletion of these metabolites from the metabolome in the murine cecum.

## Discussion

Members of the class *Clostridia* perform critical functions within the mammalian gut, including maintenance of hypoxia within the colon [18], providing colonization resistance to enteric pathogens [23, 27], and contributing to the immune education of the mammalian host organism [28]. It is known that *Clostridia* and their sister group, the *Erysipelotrichia*, are producers of short-chain fatty acids in the gut, including butyrate [13, 14]. We observed a significant decrease in most organic acids in the gut metabolome following streptomycin treatment, and an increase when *Clostridia* and *Erysipelotrichia* were given to germ-free mice. It is thus possible that these organic acids represent *Clostridia-* and *Erysipelotrichia*-derived fermentation products, but the un-targeted metabolomics approach performed in this study was unable to detect short-chain fatty acids, because of poor retention and separation of these metabolites in the columns used. We thus focused our analysis on metabolites depleted by *Clostridia* and *Erysipelotrichia*, which was indicative of nutrients consumed by members of these taxa. Notably, our data suggested that when commensal *Clostridia* and *Erysipelotrichia* were depleted in the murine large bowel, most of the detected sugar alcohols and sugar acids significantly increased, whereas when germ-free mice were associated with a defined community of 14 human *Clostridia* and 3 human *Erysipelotrichia* isolates, the inverse held true. Furthermore, *Clostridia* and *Erysipelotrichia* isolates (i.e., strains 1, 3, 7, 9, and 29) were able to grow on sugar alcohols as sole carbon *sources *in vitro and had a combined relative abundance of approximately 12% within the defined community of 14 human *Clostridia* and 3 human *Erysipelotrichia* isolates, thus explaining their ability to deplete polyols in gnotobiotic mice*.*

Sugar alcohols, such as sorbitol, xylitol, or mannitol, are of interest because of their use as low calorie sweeteners in foods [29]. They belong to a class of sugars known as polyols, which fall under the umbrella of fermentable dietary oligosaccharides, disaccharides, monosaccharides, and polyols (FODMAPs) that are poorly absorbed in the small intestine [30]. As humans lack active transporters for sugar alcohols in the small intestine, transport relies on passive diffusion, which is why these nutrients are poorly absorbed in the small intestine [30]. As a result, the bulk of dietary sugar alcohols reaches the colon, where they are fermented by the microbiota into short-chain fatty acids [31]. Sugar alcohols are naturally occurring in many fruits and vegetables, making them a prevalent constituent of the human diet [32], but excessive consumption is associated with transient bloating or diarrhea [33]. Excessive consumption of sugar alcohols also is associated with enhanced gas production and diarrhea in patients with irritable bowel syndrome [34, 35] and a diet low in FODMAPs alleviates symptoms in these patients [36]. Our results show that a *Clostridia* species, *A. caccae*, that was able to grow on sorbitol in vitro, was able to deplete this polyol when it was provided to antibiotic-treated mice as a dietary supplement.

Interestingly, a previous study suggests that sugar alcohols could be a growth factor for opportunistic enteric pathogens. *Clostridioides difficile* (*C. difficile*) is an opportunistic pathogen within the class *Clostridia*, which causes pseudomembranous colitis in individuals undergoing antibiotic therapy [37]. The metabolome of mice treated with the broad-spectrum antibiotic cefoperazone contains a higher abundance of sugar alcohols relative to the metabolome of mock treated animals, similar to what we observed after streptomycin treatment, and this treatment renders mice susceptible to *C. difficile* infection [38]. Additionally, the sugar alcohols sorbitol and mannitol are sufficient for *C. difficile* growth in NCDM in vitro [38] and increase toxin production [39]. Furthermore, depletion of alcoholic sugars by the gut microbiota contributes to colonization resistance against *C. difficile* [40]. Similarly, colonization resistance against opportunistic *Escherichia coli* infection is lost when mice receive supplementation with galacitol, a sugar alcohol [41]. Thus, depletion of sugar alcohols by endogenous *Clostridia* and *Erysipelotrichia* species might contribute to colonization resistance against opportunistic enteric pathogens.

## Conclusions

Our results identify members of the classes *Clostridia* and *Erysipelotrichia* as the main taxa in the gut microbiota that consume dietary FODMAPs during homeostasis. This finding points to *Clostridia* and *Erysipelotrichia* as a possible cause of transient bloating observed after excessive consumption of FODMAPs.

## Materials and methods

### Bacterial strains and culture techniques

A community of 14 human *Clostridia* isolates and 3 human *Erysipelotrichia* isolates was kindly provided by K Honda [21, 42], and bacteria were cultured as described previously [25].

### Animal experiments

Female C57BL/6 mice, aged 8 weeks, were obtained from The Jackson Laboratory (C57BL/6 J mice) and maintained on gamma irradiated Teklad global 18% protein rodent diet 2918 (Envigo). C57BL/6 mice were treated with 20 mg/animal of streptomycin via oral gavage or mock treated and sacrificed 3 days post treatment, and cecal contents were collected, freeze-dried, flash frozen in liquid nitrogen, and stored at − 80 °C until further processing for metabolomics. Proximal colon contents were collected, snap frozen, and stored at − 80 °C until further processing for DNA isolation and sequencing.

#### Sorbitol supplementation

C57BL/6 mice were maintained on gamma irradiated Teklad Custom Diet TD.110675 (Envigo) and treated with 20 mg/animal of streptomycin via oral gavage or mock treated. One day later, mice received drinking water supplemented with 5% sorbitol or no supplementation and were inoculated with 200 μl of a *Clostridia* culture by oral gavage. Cecal contents were collected 3 days after streptomycin treatment.

#### Microbiota transfer

Male and female germ-free Swiss-Webster mice were bred in house using gamma-irradiated chow (Purina Diet 5066; Charles River) and used for the experiment at 8 weeks of age. For transfer of human *Clostridia* and *Erysipleotrichia* strains into germ-free mice, each strain was individually inoculated into Egerth Gagnon medium and incubated for 48 h. A mixture of equal volumes for each strain was prepared, and each animal was given 200 μl of inoculum via oral gavage [25]. For microbiota transfer from conventional C57BL/6 J mice, the entire cecal content of a mouse was collected into 5 mL pre-reduced sterile PBS inside an anaerobic chamber, homogenized by vortexing and set down for 10 min to let particles settle. Immediately after, 200 μl of the homogenate supernatant was used to inoculate germ-free Swiss-Webster mice by oral gavage. For the duration of the experiment, mice were kept in sterile cages inside a biological safety cabinet. The mice were sacrificed 5 days after inoculation, and cecal contents were collected, freeze-dried, flash frozen in liquid nitrogen, then stored at − 80 °C until further processing for metabolomics; proximal colon content was collected, flash frozen in liquid nitrogen, then stored at − 80 °C until further processing for DNA isolation and sequencing.

### In vitro growth assay of *Clostridia* and *Erysipleotrichia* isolates

To assess the ability of the *Clostridia* and *Erysipleotrichia* consortium to grow on various individual carbohydrates, we used no carbon defined media (NCDM), which is a modified form of no carbon minimal media (NCMM) [38], but NCDM utilizes below vitamins and amino acids at the following concentrations: thiamine, pantothenate, nicotinic acid, riboflavin, pyridoxine, p-aminobenzoic acid, folic acid, biotin, and vitamin B12 were added in the form of ATCC Vitamin Supplement (ATCC, Manassas, Virginia) to a final concentration of 5% (v/v). Calcium, magnesium, manganese, iron, and cobalt salts were added in the form of ATCC Trace Mineral Supplement (ATCC, Manassas, Virginia) to a final concentration of 2% (v/v). Indicated carbohydrates were used at a final concentration of 0.5% (w/v). We further modified NCDM to accommodate slower growing strains by using Bacto Casamino acids (ThermoFisher) at a concentration of 4.575 g/L, supplemented with cysteine (400 mg/L), methionine (27 mg/L), alanine (72 mg/L), and tryptophan (30 mg/L). Vitamin K2 was added at a concentration of 200 μL/L. Individual strains were grown anaerobically in a volume of 2 mL of above medium containing glucose for 24 h. The entire culture volume was then spun down at 12,500 rpm for 3 min, the supernatant was discarded, and the bacterial pellet was resuspended in 750 μL of reduced PBS. Twenty-five microliters of the resuspended pellet of each strain was then used to inoculate 2-mL volumes of NCDM containing D-gluconic acid, glucose, *myo*-inositol, D-mannitol, D-sorbitol, xylitol, or no added carbohydrates in quadruplicate. After 72 h of incubation at 37 °C, growth was assessed by removing the cultures from the anaerobic chamber and reading the optical density at 600 nm. Un-inoculated NCDM was used as a blank reference.

### 16S rRNA gene amplicon sequencing sample preparation, library preparation, and sequencing

Nucleic acid extraction was done using the DNeasy Blood & Tissue Kits from Qiagen. DNA concentration was recorded using a Qubit a 2.0 fluorometer. Samples were normalized to 20 ng/μL before library preparation. The 16sV4 Region was amplified with the 515f-806R primer pair. Each 10 μL amplicon-PCR reaction consisted of 7 μL of TailorMix 2X SYBR Green qPCR Master Mix (SeqMatic, Fremont, CA, USA), 1 μL of 10 μM of each primer, and 1 μL of DNA template. Each sample was denatured at 95 °C for 10 min before undergoing 25 cycles of 95 °C for 15 s, 60 °C for 1 min and 72 °C for 1.5 min and a final extension at 72 °C for 10 min. The amplicons were pooled, purified with the DNA/RNA Purification Beads (SeqMatic, Fremont, CA, USA), and resuspended in 30 μL of 10 mM Tris, pH 8.5. The final purified library was quantified with the 2200 TapeStation (Agilent, Santa Clara, CA, USA). The Purified Library pool was quantified using Qubit and 2200 Tapestation and then diluted to 20 pM. The pool was then denatured at 94 °C on the MJ Research PTC-200 Thermal Cycler and hybridized to Illumina’s HT1 buffer. Final quantification was done using qPCR on the Roche Lightcycler 96 system. Using results from the final qPCR, the pool was loaded into a 300 cycle Illumina MiSeq Cartridge at 5 pM and sequenced to generate 150 paired-end reads. Sequencing was performed via Illumina MiSeq.

For 16S rRNA amplicon library preparation and sequencing of colon contents from germ-free mice inoculated with the 14 human *Clostridia* isolates and 3 human *Erysipelotrichia* isolates, Primers 319F (TCGTCGGCAGCGTCAGATGTGTATAAGAGACAG(spacer)*GTACTCCTACGGGAGGCAGCAGT*) and 806R (GTCTCGTGGGCTCGGAGATGTGTATAAGAGACAG(spacer)*CCGGACTACNVGGGTWTCTAAT* were used to amplify the V3-V4 domain of the 16S rRNA using a two-step PCR procedure. In step one of the amplification procedure, both forward and reverse primers contained an Illumina tag sequence (bold), a variable length spacer (no spacer, C, TC, or ATC for 319F; no spacer, G, TG, ATG for 806R) to increase diversity and improve the quality of the sequencing run, a linker sequence (italicized), and the 16S target sequence (underlined). Each 25 μl PCR reaction contained 1 Unit Kapa2G Robust Hot Start Polymerase (Kapa Biosystems), 1.5 mM MgCl_2_, 0.2 mM final concentration dNTP mix, 0.2 μM final concentration of each primer, and 1 μl of DNA for each sample. PCR conditions were an initial incubation at 95 °C for 3 min, followed by 25 cycles of 95 °C for 45 s, 50 °C for 30 s, 72 °C for 30 s and a final extension of 72 °C for 3 min. In step two, each sample was barcoded with a unique forward and reverse barcode combination using forward primers (AATGATACGGCGACCACCGAGATCTACACNNNNNNNNTCGTCGGCAGCGTC) with an Illumina P5 adapter sequence (bold), a unique 8 nt barcode (N), a partial matching sequence of the forward adapter used in step one (underlined), and reverse primers (CAAGCAGAAGACGGCATACGAGATNNNNNNNNGTCTCGTGGGCTCGG)) with an Illumina P7 adapter sequence (bold), unique 8 nt barcode (N), and a partial matching sequence of the reverse adapter used in step one (underlined). The PCR reaction in step two contained 1 Unit Kapa2G Robust Hot Start Polymerase (Kapa Biosystems), 1.5 mM MgCl_2_, 0.2 mM final concentration dNTP mix, 0.2 μM final concentration of each uniquely barcoded primer, and 1 µl of the product from the PCR reaction in step one diluted at a 10:1 ratio in water. PCR conditions were an initial incubation at 95 °C for 3 min, followed by 8 cycles of 95 °C for 30 s, 58 °C for 20 s, 72 °C for 20 s, and a final extension of 72 °C for 3 min.

The final product was quantified on the Qubit instrument using the Qubit Broad Range DNA kit (Invitrogen), and individual amplicons were pooled in equal concentrations. The pooled library was cleaned utilizing Ampure XP beads (Beckman Coulter), then the band of interest was further subjected to isolation via gel electrophoresis on a 1.5% Blue Pippin HT gel (Sage Science). The library was quantified via qPCR followed by 300-bp paired-end sequencing using an Illumina MiSeq instrument in the Genome Center DNA Technologies Core, University of California, Davis.

### 16S rRNA gene amplicon sequencing analysis

Sequencing reads were demultiplexed using QIIME 1.8 [43], and non-biological nucleotides were trimmed using Trimmomatic [44]. 16S rRNA sequencing reads were subsequently processed and assembled into amplicon sequence variants (ASV) using dada2 [45] in R. First, reads with more than 2 expected errors were removed. Dereplication and sample inference were then performed on forward and reverse reads, prior to merging. A sequence table was constructed from merged reads, and chimeric reads were subsequently removed. Taxonomy was assigned to reads to the genus level using the dada2 formatted rdp training dataset 14 which can be found here: https://zenodo.org/record/158955#.XJqlnxNKjUI. The R package phyloseq [46] was then used in downstream analysis of the data, including the generation of a phyloseq object, relative abundance bar plots, and the principle coordinate analysis plot. For the weighted unifrac analysis, the R package msa [47] was used to generate a multiple sequence alignment from the assembled reads with the following parameters: method = “ClustalW”, type = “dna”, order = “input”. The R package phangorn [48] was used to generate a maximum likelihood tree from the sequence alignment using a general time reversible (GTR) model with the following parameters: model = “GTR”, optInv = TRUE, optGamma = TRUE, rearrangement = “stochastic”, control = pml.control(trace = 0). The R package vegan was used to perform permanova analysis on weighted unifrac distances with default parameters and the R package ggplot2 was used to graph boxplots of the weighted unifrac distances. The diversity pie chart was generated by computing the ratio of ASVs within each taxonomic class level present to the total number using the R package plyr and then graphed with ggplot2. The relative abundance pie chart was generated by using phyloseq and then graphed using ggplot2. For linear discriminant analysis, data were reformatted in R, written to a tab separated text file, and then uploaded to the LEfSe galaxy server [49] where the default statistical parameters were used in the analysis to generate LDA scores and the LDA cladogram. Differential abundance analysis of taxa was performed using the R package deseq2 [25516281] with the parameters: test = “Wald”, fitType = “parametric”, cooksCutoff = FALSE. The R package omu [50] was then used to generate fold change frequency tables and differential abundance bar plots from the deseq2 modeled data.

For 16S rRNA sequencing analysis of colon contents from ex-germ-free mice inoculated with the 14 human *Clostridia* isolates and 3 human *Erysipelotrichia* isolates, reads were assigned strain level taxonomy by creating a local nucleotide blast database of the corresponding genomes using command line blast and then querying each of the 26 ASVs against the database. ASVs that matched the same strain (due to dada2 identifying multiple 16S gene copies from the same taxon as unique ASVs) were aggregated prior to the construction of the abundance boxplot using a combination of phyloseq and ggplot2.

### Phylogeny of human *Clostridia* and *Erysipelotrichia* isolates

The genome for strain 4 was omitted from this analysis because the sequence provided in NCBI was flagged as chimeric. Analysis of the chimeric sequence of strain 4 suggested that it was derived from two strains belonging to the same species, *Hungatella hathewayi.* To determine the taxonomy of the remaining 13 *Clostridia* strains and 3 *Erysipelotrichia* strains, we employed a phylogenetic approach. Sixty-three *Clostridia* and *Erysipelotrichia* genomes were curated, along with 3 *Alicyclobacilus* genomes that were included as an outgroup (Supplementary Table 6). Protein alignments of the 66 curated genomes, along with the 16 strains from the defined community of *Clostridia* and *Erysipelotrichia* isolates, were generated using clustal omega [51] for the following single copy genes: 30S ribosomal protein S3, 30S ribosomal protein S7, 30S ribosomal protein S9, 30S ribosomal protein S10, 30S ribosomal protein S12, 30S ribosomal protein S13, 30S ribosomal protein S17, 50S ribosomal protein L3, 50S ribosomal protein L14, 50S ribosomal protein L15, and 50S ribosomal protein L16. Alignments were then concatenated, providing a total alignment length of 1822 amino acids. We then used RaxML [52], with the PROTGAMMAWWAG rate of heterogeneity model to generate 20 maximum likelihood trees in order to determine the best scoring tree. Non-parametric boostrapping was subsequently performed to determine the confidence of branch placements. One hundred fifty bootstraps were sufficient for the model to reach convergence. Bootstraps were then used to draw bipartitions to the best fit tree determined by the maximum likelihood model. The tree was imported into R and visualized using the package ggtree [53].

### GC-TOF mass spectrometry

Data were acquired using the following chromatographic parameters, with more details to be found in [54]. Column: Restek corporation Rtx-5Sil MS (30 m length × 0.25 mm internal diameter with 0.25-μm film made of 95% dimethyl/5%diphenylpolysiloxane). Mobile phase: Helium. Column temperature: 50–330 °C. Flow rate: 1 mL min^−1^. Injection volume: 0.5 μL. Injection: 25 splitless time into a multi-baffled glass liner. Injection temperature: 50 °C ramped to 250 °C by 12 °C s^−1^. Oven temperature program: 50 °C for 1 min, then ramped at 20 °C min^−1^ to 330 °C, held constant for 5 min. The analytical GC column is protected by a 10-m long empty guard column which is cut by 20-cm intervals whenever the reference mixture QC samples indicate problems caused by column contaminations. We have validated that at this sequence of column cuts, no detrimental effects are detected with respect to peak shapes, absolute or relative metabolite retention times or reproducibility of quantifications. We use automatic liner exchanges after each set of 10 injections which we could show to reduce sample carryover for highly lipophilic compounds such as free fatty acids. Mass spectrometry parameters are used as follows: a Leco Pegasus IV mass spectrometer is used with unit mass resolution at 17 spectra s^−1^ from 80–500 Da at − 70 eV ionization energy and 1800 V detector voltage with a 230 °C transfer line and a 250 °C ion source.

### Metabolomics data processing

Raw data files are preprocessed directly after data acquisition and stored as ChromaTOF-specific *.peg files, as generic *.txt result files and additionally as generic ANDI MS *.cdf files. ChromaTOF vs. 2.32 is used for data preprocessing without smoothing, 3 s peak width, baseline subtraction just above the noise level, and automatic mass spectral deconvolution and peak detection at signal/noise levels of 5:1 throughout the chromatogram. Apex masses are reported for use in the BinBase algorithm. Result *.txt files are exported to a data server with absolute spectra intensities and further processed by a filtering algorithm implemented in the metabolomics BinBase database. The BinBase algorithm (rtx5) used the settings: validity of chromatogram (< 10 peaks with intensity > 10^7^ counts s^−1^), unbiased retention index marker detection (MS similarity > 800, validity of intensity range for high m/z marker ions), and retention index calculation by 5th order polynomial regression. Spectra are cut to 5% base peak abundance and matched to database entries from most to least abundant spectra using the following matching filters: retention index window ± 2000 units (equivalent to about ± 2 s retention time), validation of unique ions and apex masses (unique ion must be included in apexing masses and present at > 3% of base peak abundance), mass spectrum similarity must fit criteria dependent on peak purity and signal/noise ratios and a final isomer filter. Failed spectra are automatically entered as new database entries if s/n > 25, purity < 1.0, and presence in the biological study design class was  > 80%. All thresholds reflect settings for ChromaTOF v. 2.32. Quantification is reported as peak height using the unique ion as default, unless a different quantification ion is manually set in the BinBase administration software BinView. A quantification report table is produced for all database entries that are positively detected in more than 10% of the samples of a study design class (as defined in the miniX database) for unidentified metabolites. A subsequent post-processing module is employed to automatically replace missing values from the *.cdf files. Replaced values are labeled as ‘low confidence’ by color coding, and for each metabolite, the number of high-confidence peak detections is recorded as well as the ratio of the average height of replaced values to high-confidence peak detections. These ratios and numbers are used for manual curation of automatic report data sets to data sets released for submission. These data were then normalized to the mTIC value (sum of the peak heights of the known metabolites).

### Metabolomics data analysis

Metabolomics data were loaded into R, and the following analysis was performed using the R package omu [50]. Compound hierarchy was assigned from the KEGG [55] hierarchy databases in omu’s system data. The data were normalized using the natural log, Student’s *t* test was performed to calculate *p* values, a Benjamini–Hochberg procedure was used to perform false discovery rate correction for type 2 errors, and log2 fold change of group means was calculated to visualize effect size. Fold change frequency tables were generated to make bar plots. Volcano plots were generated using FDR corrected *p* values and log2 fold change between group means. Principle component analysis was performed on natural log transformed data.

### D-sorbitol measurement

Mouse cecal contents were homogenized in 1 mL of sterile PBS, centrifuged at 300* g* for 10 min at 4 °C, and then the supernatants were collected. The supernatants were filtered through a 10-kDa spin column (Biovision, Milpitas, CA). D-Sorbitol concentrations were determined by D-sorbitol assay kit (Biovision, Milpitas, CA) according to the manufacturer’s instruction.

## Supplementary Information

**Additional file 1.**

**Additional file 2.**

**Additional file 3: Figure S1.** Amplicon sequence variants (ASVs) belonging to *Erysipelotrichia *which significantly changed abundance after antibiotic treatment (related to Fig. 2). Mice were mock-treated (*n* = 8) or received a single dose of streptomycin (*n* = 8) and bacterial communities in the colon contents were analyzed 3 days later. Box plots showing the relative abundance of *Erysipelotrichia *ASVs in mock-treated and streptomycin-treated mice.

**Additional file 4.**

**Additional file 5.**

**Additional file 6.**

**Additional file 7.**

## Data Availability

Illumina sequences obtained in the present study were deposited in the Sequence Read Archives (SRA) NCBI database under BioProject ID: PRJNA560082. Untargeted metabolomics data are provided in Supplementary Tables 1, 3 and 4.
