# Supplementary material for: The metabolic footprint of Clostridia and Erysipelotrichia reveals their role in depleting sugar alcohols in the cecum
Source: Microbiome. 2021 Aug 19;9:174. doi: 10.1186/s40168-021-01123-9 (PMC8375055; doi:10.1186/s40168-021-01123-9)
Supplement: Supplementary file 4 — Additional file 3: Figure S1. Amplicon sequence variants (ASVs) belonging to Erysipelotrichia which significantly changed abundance after antibiotic treatment (related to Fig. 2). Mice were mock-treated (n = 8) or received a single dose of streptomycin (n = 8) and bacterial communities in the colon contents were analyzed 3 days later. Box plots showing the relative abundance of Erysipelotrichia ASVs in mock-treated and streptomycin-treated mice. [file 40168_2021_1123_MOESM4_ESM.pdf]

SUPPLEMENTARY FIGURES

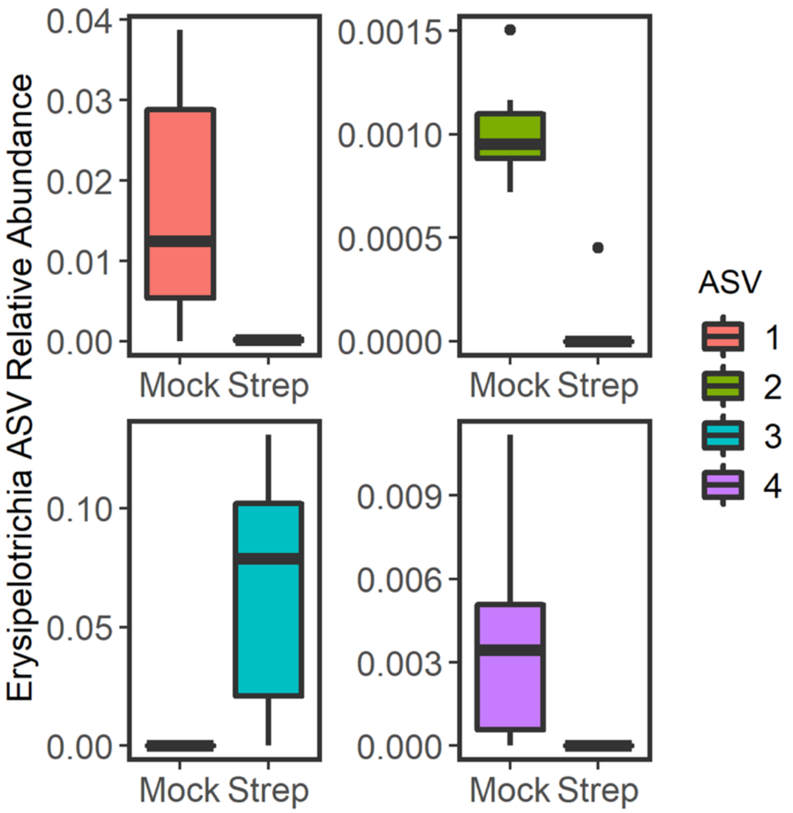

Figure S1: Amplicon sequence variants (ASVs) belonging to *Erysipelotrichia* which significantly changed abundance after antibiotic treatment (related to Figure 2). Mice were mock-treated (n = 8) or received a single dose of streptomycin (n = 8) and bacterial communities in the colon contents were analyzed three days later. Box plots showing the relative abundance of *Erysipelotrichia* ASVs in mock-treated and streptomycin-treated mice.
